# Development of a database and analytical environment for precision quality management in high‐precision radiotherapy

**DOI:** 10.1002/acm2.70324

**Published:** 2025-11-05

**Authors:** Taichi Wada, Kazunori Miyaura, Kouzou Murakami, Yoshikazu Kagami

**Affiliations:** ^1^ Graduate School of Health Sciences Showa Medical University Tokyo Japan; ^2^ Department of Radiation Oncology, School of Medicine Showa Medical University Tokyo Japan

**Keywords:** database, gamma analysis, high‐precision radiotherapy, patient‐specific quality assurance, TG‐218

## Abstract

**Background:**

High‐precision radiotherapy, especially intensity‐modulated radiotherapy (IMRT), requires stringent quality assurance (QA) owing to its complexity. Moreover, patient‐specific QA (PSQA) is essential to ensure accurate dose delivery.

**Purpose:**

This study aimed to develop a centralized PSQA database and analytical environment to improve consistency, efficiency, and safety in QA processes across institutions.

**Methods:**

A relational database was implemented using MOSAIQ Oncology Analytics, integrating structured input forms, statistical process control, and visualization tools. PSQA data from four institutions were collected and analyzed based on AAPM TG‐218.

**Results:**

A total of 835 PSQA cases were analyzed across five disease sites. Statistically derived action and tolerance limits revealed interfacility variability. Visualization dashboards enabled detection of trends and outliers, supporting real‐time QA monitoring.

**Conclusions:**

The system enhanced QA consistency, enabled benchmarking, and supported data‐driven improvements. Centralized QA databases are effective tools for advancing radiotherapy quality and safety.

## 1 INTRODUCTION

Cancer remains a significant global public health challenge,^1^ and radiotherapy is a cornerstone treatment modality alongside surgery and chemotherapy.^2^ In Japan, the Ministry of Health, Labor and Welfare reported in its 2022 mortality statistics that malignant neoplasms account for >24% of annual deaths,^1^ highlighting the urgency of effective treatment strategies. Therefore, high‐precision radiotherapy technologies, such as intensity‐modulated radiotherapy (IMRT), have revolutionized clinical outcomes.^3^, ^4^ IMRT enables clinicians to deliver nonuniform radiation intensities across multiple beam angles, resulting in highly conformal dose distributions that better spare healthy tissue. However, the complexity of IMRT necessitates stringent quality assurance (QA) protocols to ensure that each patient receives treatment as planned.^5^, ^6^

Among the various QA practices implemented in modern radiation oncology, patient‐specific QA (PSQA) is considered indispensable.^6^, ^7^ PSQA is designed to detect discrepancies between the planned and delivered dose distributions before treatment is initiated, thereby reducing the likelihood of adverse events caused by incorrect beam configurations or system errors. Alarmingly, data from the World Health Organization's Radiotherapy Risk Profile indicate that more than half of the reported radiotherapy incidents are caused by errors during the planning or delivery stages.^2^

Despite the vital role of PSQA, its implementation is often hindered by outdated or inconsistent data management practices. In many facilities, results are recorded manually using spreadsheets or static PDF reports.^8^ These decentralized methods restrict the aggregation, comparison, or analysis of data systematically. Furthermore, the lack of standardization complicates the identification of trends and anomalies, and human error in data transcription further undermines reliability.^9^, ^10^

To address these challenges, this study focused on the development and implementation of a relational database and analytical environment tailored to PSQA in high‐precision radiotherapy.^7^, ^11^, ^12^, ^13^ The goal was to facilitate centralized data collection, streamline QA workflows, enable robust statistical analyses, and support real‐time visualization. Thus, the system aims to promote uniform QA practices across multiple facilities and contribute to continuous improvements in treatment quality and patient safety.^13^, ^14^ Although the term “IMRT” is used throughout this manuscript to maintain consistency with TG‐218 and related QA guidelines, most participating institutions primarily use VMAT, a technically advanced rotational delivery technique evolved from IMRT. Accordingly, the QA records analyzed in this study, including those labeled as “VMAT Verification Sheets,” predominantly reflect VMAT‐based treatments. Therefore, the study's methodology and findings are directly relevant and applicable to VMAT as the primary treatment modality.

## 2 METHODS

A custom relational database was designed and implemented using MOSAIQ Oncology Analytics (MOA), a commercial platform commonly used for clinical data integration in oncology. The MOA was selected for its ability to manage structured data, perform statistical calculations efficiently, and integrate seamlessly into existing clinical workflows. The database was structured to accommodate multiple data streams, including numerical measurements, categorical classifications, and time‐stamped metadata from various radiotherapy centers within the Showa Medical University Group.

To support retrospective and prospective analyses of PSQA implementation records, a dedicated PSQA data input form was created within MOSAIQ. This form captured relevant clinical and QA data directly into the system and enabled standardized data collection across institutions. A total of 835 PSQA records were accumulated from January 2016 onward and integrated within the MOA environment for analysis.

Data were collected from QA forms such as the “VMAT Verification Sheet” and “Radixact QA Record.” These included patient identifiers (not anonymized), QA measurement and plan approval dates, measured versus calculated absolute dose values,^10^, ^15^ and gamma‐passing rate results under multiple criteria (e.g., 3%/3 mm and 2%/2 mm).^16^, ^17^, ^18^, ^19^ However, the 3%/2 mm criterion recommended by TG‐218 was not uniformly implemented across participating institutions during the data collection period. Consequently, action limits (AL) and tolerance limits (TL) were primarily calculated using the 3%/3 mm criterion, with supplementary analyses performed at 2%/2 mm when available.

All records were linked via patient ID as the primary key, enabling longitudinal tracking of QA performance. Patient IDs remained securely stored within institutional networks, with access strictly limited in accordance with institutional privacy policies.

Terminology and formatting inconsistencies across facilities were resolved through data curation. Disease classification was normalized and included as a mandatory field for condition‐specific analyses. Data validation rules were applied during entry, including range checks for numerical fields and controlled vocabularies for categorical fields.

To minimize transcription errors and improve consistency in data entry, structured input forms were developed within the MOSAIQ interface. These forms included drop‐down menus for device types and anatomical sites, and employed automated flagging for missing or out‐of‐range values.

The tolerance width ΔA, which represents the difference between the upper and lower intervention thresholds, was calculated using the following equation:

(1) $$\Delta A\;=\beta \sqrt{\sigma^{2}+{\left(\bar{x}-T\right)}^{2}}$$

where, β is a constant, σ is the standard deviation of the process (e.g., the gamma‐passing rate), x̄ is the process mean, and T is the process target value (commonly set to 100% or 0%).

Based on this value, the AL, which indicates the lower threshold for acceptable QA results, was defined as:

(2) Actionlimits=100−ΔA/2

To monitor process stability over time, statistical process control was implemented using moving range control charts. The upper and lower control limits (UCL and LCL, respectively) were calculated as:

(3) $$\mathrm{UCL}\;=\;\mathrm{center}\;\mathrm{line}+2.660\cdot \bar{\mathrm{mR}}$$

(4) $$\mathrm{LCL}\;=\;\mathrm{center}\;\mathrm{line}-2.660\cdot \bar{\mathrm{mR}}$$

Here, the center line and Here, the center line is defined as follows:

(5) $$\mathrm{center}\;\mathrm{line}=\frac{1}{2}\sum_{1}^{n}x$$

The average moving range is calculated as follows:

(6) $$\mathrm{Moving}\;\mathrm{Range}\;\bar{\mathrm{mR}}=\frac{1}{n-1}\sum_{i\;=2}^{n}\left|x_{i}-x_{i-1}\right|$$

This approach allows the early detection of trends or deviations in QA metrics that may require intervention.^20^

Database Configuration and Multi‐Institutional Workflow

**FIGURE 1:**
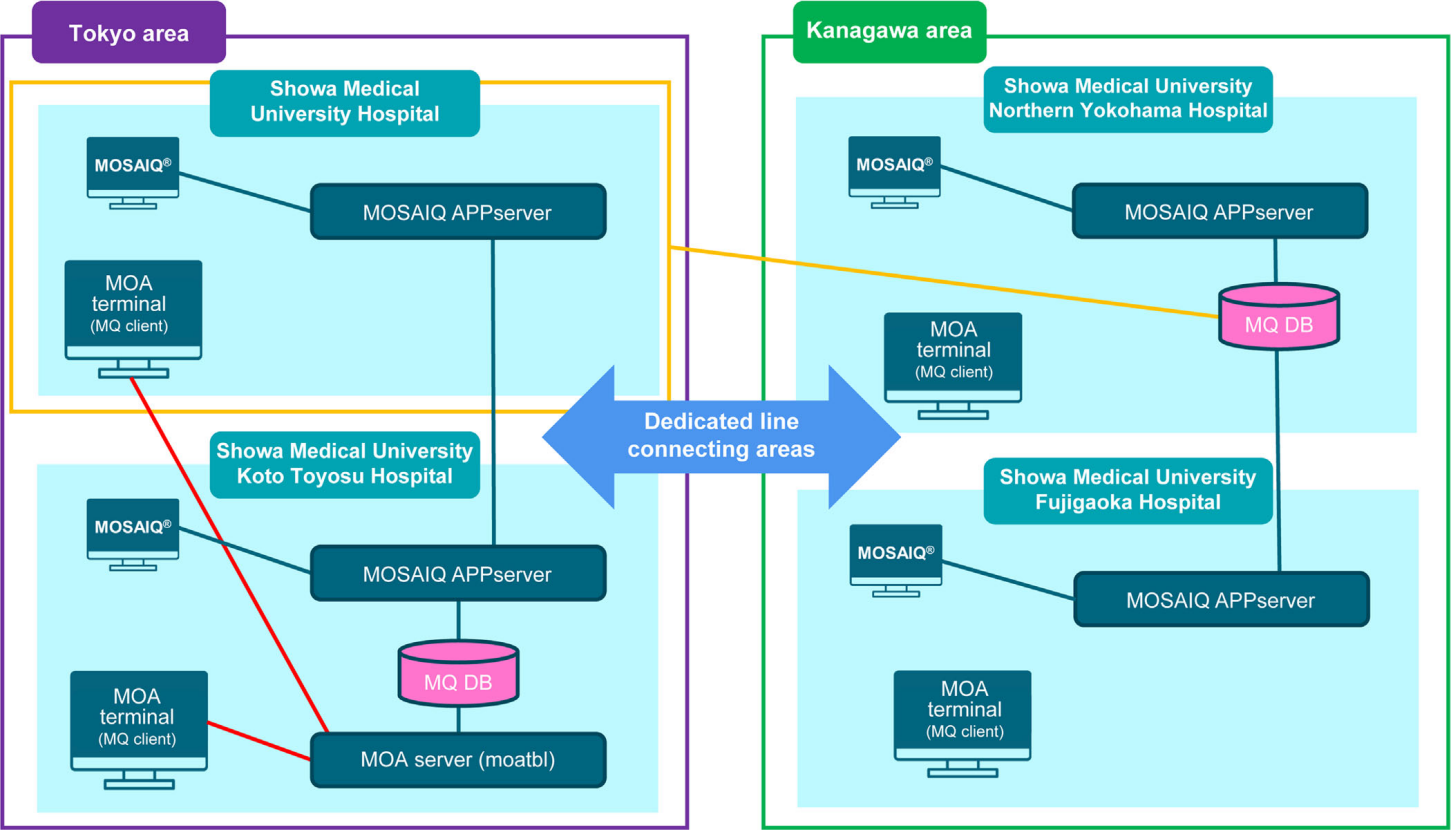
Network configuration of the multi‐institutional patient‐specific quality assurance (PSQA) system (Showa Medical University Group).

**FIGURE 2:**
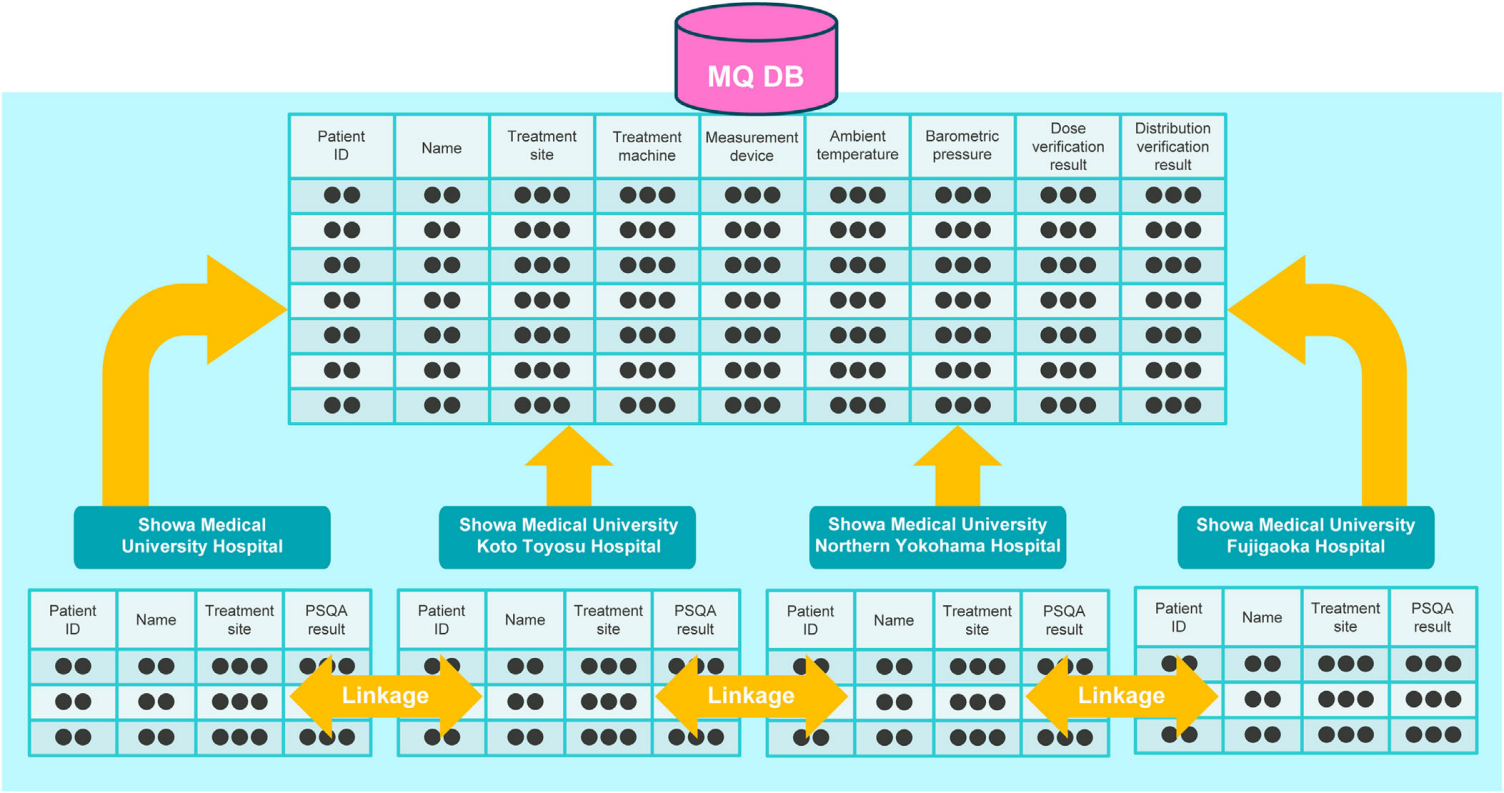
Relational database structure for centralized PSQA management.

Figures 1 and 2 illustrate the multi‐institutional configuration and database architecture of MOA as implemented in this study. A collaborative radiotherapy network was established across four participating institutions, each operating its own MOSAIQ system. In one region, data from multiple sites were securely transmitted to a centralized MOA server via intra‐group connections. A similar configuration was applied in another region, enabling reliable data exchange across geographically dispersed centers (Figure 1).

The database was structured as a relational system linking PSQA measurements with clinical metadata such as treatment site, device model, and patient ID. As depicted in Figure 2, records were normalized and connected via patient ID keys (securely stored within institutional networks) to reduce redundancy and support cross‐facility analysis. This structure facilitates efficient data aggregation and querying within a shared MOA environment (Figure 2).

In this configuration, “real‐time visualization” refers to automatic updates of QA dashboards each time a user initiates a search or applies filters. Although the system does not update continuously, it dynamically reflects the most recent data available at the time of interaction, ensuring near‐real‐time feedback. Access to dashboards is restricted to authorized users through virtual private network (VPN)‐secured connections, with no additional client software or licensing required. A data analysis worksheet was created within the MOA interface to enable the automated computation of TL and AL based on the TG‐218 methodology. The calculation logic was embedded in worksheet templates to instantly generate ΔA, AL, and TL values from newly entered PSQA data. This reduced human error. In this study, a collaborative radiotherapy network was established involving four participating institutions, each operating its own MOSAIQ system. PSQA data were entered into standardized input forms within MOSAIQ and transmitted via APP servers to a centralized MOA server for integrated analysis.

The database was structured as a relational system, linking QA records through patient IDs used as primary keys. Although anonymization was not performed, patient identifiers were securely stored and managed within institutional networks under strict access controls.

Within the MOA platform, data aggregation and statistical calculations are automatically performed whenever a user initiates a query using defined filters or conditions. Although updates are not continuous, the system delivers near‐real‐time visualization by dynamically reflecting the most current data at the time of interaction.

MOA dashboard access is limited to authorized users via secure VPN connections from participating sites. No additional client software or licensing is required, allowing for secure, centralized data management and analysis across facilities using existing infrastructure.

**FIGURE 3:**
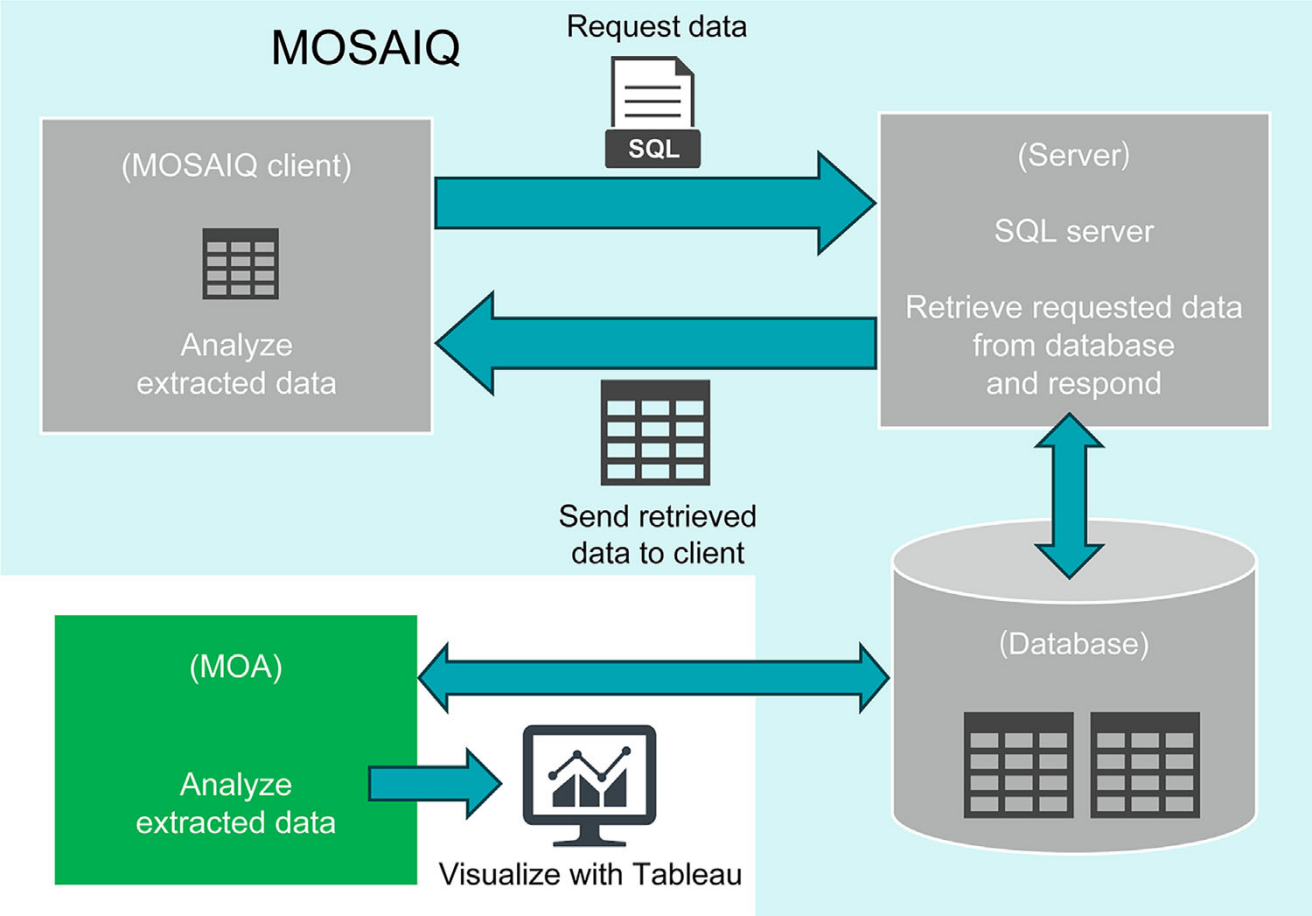
Data flow and visualization framework in the centralized PSQA system.

These computed values were then visualized using Tableau, which provides dynamic, interactive dashboards for trend analysis, facility comparisons, and temporal pattern recognition (Figure 3).

The dashboard incorporated multilevel filters—including treatment site, time window, gamma criteria, and machine model—allowing users to drill down into specific data subsets for detailed evaluation.

In this context, “real‐time visualization” refers to the dashboard's ability to update automatically whenever a user runs a query or applies filters. Although the system does not perform continuous automatic updates, it dynamically displays the most recent data at the time of user interaction, providing near‐real‐time feedback.

Outlier detection was implemented using the interquartile range method. Abnormal data entries were flagged in real time, and alerts were automatically triggered when calculated AL or TL thresholds were breached. This supported timely intervention and enabled continuous QA monitoring.

PDF reports were generated in compliance with medical documentation standards. These included summaries of gamma‐passing rates, TL and AL calculations, and flagged entries. Reports were appended to patient records or reviewed by QA committees during audits.^20^

Four Showa Medical University‐affiliated hospitals participated in this study, each with distinct radiotherapy equipment and QA protocols (Table 1).

**TABLE 1:** Treatment machines and QA devices used by the Showa Medical University Group.

	Showa Medical University Hospital	Showa Medical University Koto Toyosu Hospital	Showa Medical University Northern Yokohama Hospital	Showa Medical University Fujigaoka Hospital
Machine	Clinac iX^a^ Radixact^b^	Infinity^c^	True Beam STx^a^	True Beam^a^
QA Device	ArcCHECK^d^(iX) Delta4^e^(Radixact) Farmer Chamber^f^(iX) Exradin A1SL ION Chamber^g^(Radixact)	ArcCHECK^d^ Farmer Chamber^f^	Delta4^e^ Pinpoint Chamber^f^	ArcCHECK^d^ Farmer Chamber^f^

^a^ Varian Medical Systems, Palo Alto, CA, USA.

^b^ Accuray Incorporated, Sunnyvale, CA, USA.

^c^ Elekta AB, Stockholm, Sweden.

^d^ Sun Nuclear Corporation, Melbourne, FL, USA.

^e^ ScandiDos AB, Uppsala, Sweden.

^f^ PTW, Freiburg, Germany.

^g^ Standard Imaging Inc., Middleton, WI, USA.

In summary, this section demonstrates the construction and implementation of a centralized automated PSQA data management and analysis system.

By integrating structured input via MOSAIQ, real‐time statistical calculations through the MOA, and dynamic visualization using Tableau, the framework ensures both operational efficiency and robust quality monitoring in high‐precision radiotherapy.

This study was approved by the Institutional Review Board of Showa University (Approval No. 2023‐052‐B; now Showa Medical University).

## 3 RESULTS

In total, 835 PSQA entries were collected from the developed relational database from January 2016 to the present. The records were categorized into five anatomical treatment sites, as follows:

‐ Central nervous system (CNS): 147 cases
‐ Head and Neck: 256 cases
‐ Chest: 89 cases
‐ Pelvis: 258 cases
‐ Others: 85 cases

**FIGURE 4:**
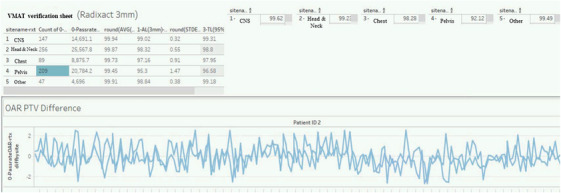
MOA search results interface displaying calculated action and tolerance levels, along with longitudinal QA trend visualization.

This robust dataset enabled detailed statistical analyses across disease categories and institutions. The gamma‐passing rate results demonstrated high consistency; however, device‐ and site‐specific variability was observed. Using the database's query function, users could designate time ranges and filtering conditions to view PSQA results (Figure 4).

The calculated AL and TL values were displayed alongside visual representations such as control charts and scatter plots, providing intuitive insight into longitudinal performance.

AL and TL values were calculated based on the process characteristics observed within the Showa Medical University Group. The gamma‐passing rate and PSQA evaluation index were 99.4% ± 1.3% at 3%/3 mm and 94.3% ± 7.7% at 2%/2 mm, respectively.

Although TG‐218 recommends the 3%/2 mm gamma criterion as a universal evaluation threshold for PSQA evaluation, all participating institutions in this study primarily used the 3%/3 mm threshold, consistent with earlier guidelines such as TG‐119. Some institutions also applied more stringent criteria such as 2%/2 mm for internal assessments; however, variability in implementation prevented uniform application across the dataset. Consequently, AL and TL values were primarily calculated using the 3%/3 mm criterion, with additional analysis at 2%/2 mm where available. In future iterations, we aim to incorporate consistent 3%/2 mm evaluations to align with TG‐218 recommendations.^21^

Although the term “IMRT” is used throughout this manuscript to align with the terminology of TG‐218 and related QA guidelines, most participating institutions primarily employ VMAT, a technically advanced rotational delivery technique derived from IMRT. Therefore, the QA records analyzed in this study, including those labeled as “VMAT Verification Sheets,” reflect VMAT‐based treatments. The methodological framework and findings are thus applicable to VMAT as the primary modality. The absolute dose difference was 0.46% ± 0.98%. These metrics were used to calculate the TL and AL values in accordance with TG‐218 recommendations.^21^ In particular, for the 3%/3 mm gamma criterion, the following limits were determined.

‐ CNS:   AL = 99.0%, TL = 99.6%
‐ Head & Neck: AL = 96.4%, TL = 97.2%
‐ Chest:   AL = 97.8%, TL = 97.2%
‐ Pelvis:  AL = 93.6%, TL = 94.7%
‐ Neck:   AL = 96.4%, TL = 97.2%

These calculated limits provided context for interpreting QA outcomes according to site and were graphically visualized using the MOA's display functionality. A similar analysis was performed for each hospital within the Showa Medical University Group^22^ (Table 2).

**TABLE 2:** PSQA result comparison for pelvis IMRT under the 3%/3 mm evaluation criterion across four facilities.

Pelvis 3%/3 mm	Showa Medical University Hospital	Showa Medical University Koto Toyosu Hospital	Showa Medical University Northern Yokohama Hospital	Showa Medical University Fujigaoka Hospital
AL (%)	93.6	97.6	99.5	88.7
TL (%)	94.7	98.1	99.7	90.3

Although all institutions achieved high gamma‐passing rates, minor discrepancies were evident. Showa Medical University Hospital demonstrated the highest uniformity, with minimal standard deviation in the results. In contrast, Showa Medical University Fujigaoka Hospital exhibited slightly lower average values and larger variability. These findings suggest that differences in machine models, QA equipment (including dosimeters, electrometers, and phantoms), calibration protocols, and data entry processes may affect consistency across sites.

Measurement uncertainties and systematic errors in dosimetry have long been recognized as sources of variability in radiation therapy, specifically when comparing across different institutions or devices.^23^

The increased standard deviation observed under the 2%/2 mm criterion further highlights its heightened sensitivity, which may be useful for detecting subtle changes in treatment delivery but requires cautious interpretation.^24^

The visualization features within the MOA system were important for identifying institutional trends and isolated anomalies. For example, a facility that frequently recorded lower gamma‐passing rates triggered a group‐wide review of the QA processes. In other instances, extreme data points were traced back to manual input errors, which were subsequently corrected via built‐in alert functions.^25^

These insights highlight the potential of a centralized, multi‐institutional QA database for continuous monitoring and feedback. The similar performance between identical machines suggests that benchmarking and process improvement can be pursued efficiently across facilities. In contrast, the deviations observed across equipment vendors emphasize the need for broader standardization in QA methodology. This supports the value of collaborative data environments for advancing the safety, precision, and reproducibility of modern radiotherapy.^26^

In summary, this section presents a comprehensive evaluation of PSQA outcomes using a centralized database system. Through the integration of statistical metrics, visualization tools, and multi‐institutional comparisons, the analysis demonstrated the system's capacity to enhance quality consistency, detect anomalies, and support benchmarking efforts across diverse radiotherapy settings.

## 4 DISCUSSION

This study demonstrated that transitioning from decentralized and manual QA processes to a centralized structured database significantly improves the efficiency and transparency of PSQA in high‐precision radiotherapy and the consistency and long‐term traceability of quality indicators.^9^ The developed system allows institutions to objectively visualize performance trends and evaluate deviations across time and between facilities. This is particularly important in the context of IMRT, where treatment complexity demands high accuracy and reproducibility in dose delivery.^21^

A notable advantage of this database system is its ability to identify both systemic and random errors by aggregating data from multiple institutions. The consistent structure of the database allowed for standardized comparison and trend analysis, highlighting facility‐specific characteristics.^10^ For instance, one center persistently recorded lower gamma‐passing rates, which, upon further investigation, were related not to hardware malfunction but to stricter local evaluation criteria and differing interpretations of measurement procedures.^13^ Such insights are difficult to obtain using conventional spreadsheet‐based methods and highlight the value of centralized QA monitoring.^25^

Furthermore, variations in passing rates, particularly under stricter gamma criteria (e.g., 2%/2 mm), reflected differences in treatment equipment and planning systems, as well as variability in staff proficiency and experience levels.^20^ In facilities where technologists had undergone more extensive QA training or participated regularly in academic QA programs, the data exhibited tighter control limits and fewer outliers. This finding supports previous studies linking staff competency and ongoing education to higher treatment accuracy and patient safety.^6^, ^20^

From a methodological perspective, the statistical modeling based on TG‐218 provided a solid foundation for defining TL and AL.^21^ By calculating ΔA, TL, and AL using objective process parameters such as standard deviation and mean deviation from the target value, the study moved beyond subjective thresholds toward a data‐driven definition of acceptable performance.^21^ This approach allows for the creation of alerts and intervention criteria tailored to each facility's actual performance history, which is more realistic than applying universal fixed thresholds.

In addition, the system employs process control methods using moving ranges and control limits (UCL/LCL), offering another layer of real‐time monitoring.^24^ This was effective for the early detection of drift or sudden changes in QA results that might otherwise go unnoticed. For instance, temporary degradation in device performance or software updates affecting dose calculations could be detected through MR‐based alerts, facilitating timely corrective action.^24^

Another key benefit of this system is its ability to facilitate collaborative QA improvement across multiple centers.^14^ With consistent data formatting and shared metrics, institutions can benchmark performance, exchange findings, and engage in peer‐supported learning. Such networks could serve as a foundation for national QA registries or multicenter clinical trials, where QA homogeneity is essential for data comparability and reproducibility.^25^

Compared with commercial QA software packages that offer database functionalities, our MOA‐based system offers notable advantages in cost‐effectiveness, workflow integration, and clinical adaptability. Since it operates on the existing MOA infrastructure already implemented across participating institutions, the system requires no additional hardware or software licenses, allowing for economical and scalable deployment.

A key strength of our system lies in its ability to integrate PSQA data with structured clinical information, including ICD‐10 codes and cancer registry records. This integration facilitates future multivariate analyses to examine relationships between QA performance and treatment complexity, supporting the development of disease‐specific AL and TL thresholds.^26^ Such comprehensive data linkage is currently not achievable with most commercial platforms, making our system a strong foundation for continuous quality improvement and clinical research.

However, several limitations were also observed. Although the system improved the data input consistency, it still relied on manual entry for many variables, including environmental conditions and disease classifications.^13^ To further minimize human error and improve efficiency, future developments should include direct device‐to‐database integration. The automated transfer of measurement data, dose calculations, and plan parameters via DICOM‐RT or vendor‐specific APIs would allow for seamless and error‐free data capture.^27^, ^28^

Furthermore, while this study primarily focused on the gamma‐passing rate as the principal QA metric, this only captures part of treatment quality. Expanding the database to incorporate additional indicators such as point dose deviations, isodose comparisons, clinical outcomes, and radiobiological indices (e.g., NTCP/TCI values) would allow for a more comprehensive evaluation of treatment effectiveness.^18^, ^29^

Finally, leveraging the growing predictive analytics dataset using machine learning may enable proactive QA.^12^ Predictive models could identify conditions likely to result in QA failures, thereby improving resource allocation and reducing the risk of undetected issues.

In summary, this section highlights the clinical and operational advantages of a centralized PSQA database system. It supports real‐time monitoring, cross‐institutional benchmarking, and evidence‐based QA practices, ultimately contributing to safer and more efficient radiotherapy.^11^

## 5 CONCLUSION

This study established Showa Medical University Group‐specific PSQA tolerance levels and demonstrated enhanced sensitivity in detecting deviations across treatment facilities. By leveraging statistically derived AL and TL based on actual process behavior, the proposed system offers a realistic and adaptive framework for QA in high‐precision radiotherapy.

Importantly, the centralized database can link with clinical information for patients undergoing radiotherapy managed within the same system, enabling multifaceted analysis that integrates QA results with clinical outcomes. This approach allows for future investigations into the correlations between QA metrics and patient safety or efficacy indicators.

Numerous benefits are anticipated from this approach, including more precise control of IMRT accuracy and safety, improved operational efficiency across departments, and more comprehensive evaluations of clinical data. As treatment modalities grow in complexity, the continued development and integration of structured QA databases will be essential in supporting high standards of care and advancing the field of radiation oncology.

## AUTHOR CONTRIBUTIONS

**Taichi Wada**: Conceptualization; data curation; formal analysis; investigation; writing—original draft. **Kazunori Miyaura**: Supervision; methodology; validation; writing—review & editing. **Kouzou Murakami**: Resources; technical support; writing—review & editing. **Yoshikazu Kagami**: Resources; clinical insight; writing—review & editing.

## CONFLICT OF INTEREST STATEMENT

The authors declare no conflicts of interest related to this work.

## Ethics Statement

This study was approved by the Institutional Review Board of Showa University (Approval No. 2023‐052‐B; now Showa Medical University).

## Data Availability

The data underlying this study contain sensitive clinical information and are not publicly available. Aggregated/derived data and analysis scripts are available from the corresponding author on reasonable request.
